# Effects of City Expansion on Heat Stress under Climate Change Conditions

**DOI:** 10.1371/journal.pone.0117066

**Published:** 2015-02-10

**Authors:** Daniel Argüeso, Jason P. Evans, Andrew J. Pitman, Alejandro Di Luca

**Affiliations:** 1 Climate Change Research Centre, University of New South Wales, Sydney, Australia; 2 ARC Centre of Excellence for Climate System Science, University of New South Wales, Sydney, Australia; University of Washington, UNITED STATES

## Abstract

We examine the joint contribution of urban expansion and climate change on heat stress over the Sydney region. A Regional Climate Model was used to downscale present (1990–2009) and future (2040–2059) simulations from a Global Climate Model. The effects of urban surfaces on local temperature and vapor pressure were included. The role of urban expansion in modulating the climate change signal at local scales was investigated using a human heat-stress index combining temperature and vapor pressure. Urban expansion and climate change leads to increased risk of heat-stress conditions in the Sydney region, with substantially more frequent adverse conditions in urban areas. Impacts are particularly obvious in extreme values; daytime heat-stress impacts are more noticeable in the higher percentiles than in the mean values and the impact at night is more obvious in the lower percentiles than in the mean. Urban expansion enhances heat-stress increases due to climate change at night, but partly compensates its effects during the day. These differences are due to a stronger contribution from vapor pressure deficit during the day and from temperature increases during the night induced by urban surfaces. Our results highlight the inappropriateness of assessing human comfort determined using temperature changes alone and point to the likelihood that impacts of climate change assessed using models that lack urban surfaces probably underestimate future changes in terms of human comfort.

## Introduction

Cities create an environment that is clearly distinct from their surrounding areas. Urban structures alter the surface energy budget [1], modify the vertical profile of various atmospheric properties, interact with both local and regional circulation, and introduce additional sources of heat (e.g. anthropogenic heat). As a result, the climate conditions in the urban environment significantly differ from their rural counterparts. The study of the urban climate has therefore attracted considerable attention from a broad range of researchers in the last few decades with considerable effort devoted to understanding the urban-atmosphere interactions under present climate [2–5]. Although the effect of urban areas is confined in spatial extent and relatively small in global terms [6, 7] it has implications for most of the world’s population. Over half of the world population currently lives in cities and urban population is expected to rapidly increase in the coming decades [8]. Studies focused on the specific changes in climate that might occur in cities are therefore crucial to explore how exposed the urban population is to future climate risks. This examination of vulnerability offers the potential to then design adequate mitigation strategies.

Few climate projections used to study future climate include urban-induced effects although some efforts have been made to incorporate urban parameterizations in global models [9–11]. These agree on the necessity to represent cities explicitly in climate simulations because the effects of urbanization cannot be simply added to the climate change signal due to the non-linear nature of their interactions [11, 12]. These studies offer valuable insight into the contrasts between urban and rural responses to climate change, but limitations exist in representing cities within Global Climate Models (GCMs) as a consequence of their coarse spatial resolution [12, 13]. For instance, GCMs do not include any spatial heterogeneity within cities because the extent of urban areas is generally smaller than a grid cell. Urban and rural areas also share the same boundary layer and differences between them are not represented. Finally, mesoscale processes linked to urban-rural interactions are not resolved.

Experiments using nested Regional Climate Models (RCMs) driven either by GCM simulations or re-analyses have been conducted to overcome the spatial scale disparity between GCMs horizontal grid spacing and urban areas. While they constitute an advance with respect to GCMs’ spatial resolution, they were either completed at resolutions still too coarse to represent processes that are crucial to describe urban climate (25km, [14]; 20km, [15]) or studied only specific seasons (3km, [16]; 20km, [17]). A recent study performed high-resolution simulations over longer periods to quantify temperature response to urban expansion and climate change [18]. Overall, the estimated contribution to future mean warming from urban structures found in previous studies was in the range 1–2°C [15] by the end of the century and 1.1–2°C for minimum temperature by 2050 [14, 18].

While most previous studies focused on near-surface temperature, some also analyzed wind, surface evaporation, and precipitation [19, 20]. Urban structures primarily affect local temperatures, which are generally warmer in cities than in surrounding areas.

Nevertheless, cities also alter near-surface humidity through changes in the surface energy partitioning [21]. Both variables play a central role in human comfort [22]. Urbanization typically acts on these two variables in opposite directions [23], increasing temperature and reducing humidity. Heat stress conditions in a changing climate were estimated combining these two variables by previous authors using either GCMs [24] or RCMs [25], but only for non-urban areas and thus without considering the unique properties of the urban environment. They found that humidity increases tend to enhance heat-stress changes due to temperature rise, especially in areas of elevated humidity such as near the tropics and along the coast.

The question of whether changes in urban humidity may partially offset heat stress induced by higher temperatures in the urban environment is important. Fischer et al. in 2012 [26] analyzed the differences between rural and urban environments for future heat stress and found substantially higher values in cities but a similar increase rate in both areas. However their study addressed the problem using a GCM, which is subject to the aforementioned limitations and thus the question remains open to a large extent. An adequate answer will help us understand impacts of climate change on urban dwellers and could lead to the design of efficient approaches to reduce their vulnerability.

In this paper, the capabilities of high-resolution (2 km) regional climate modeling were used to simulate interactions between city structures and the atmosphere in the Sydney Area, and measure the influence of climate change and urbanization on human comfort. In particular, the combined effect of changes in near-surface temperature and vapor pressure in Sydney were used to calculate a comfort index (simplified wet-bulb globe temperature) and quantify local heat stress changes due to urban expansion and climate change. Their relative contribution of changes in temperature and vapor pressure were then examined to determine which variable dominates the changes in heat stress during day and night.

The paper is structured as follows: In Section 2 the heat stress index and the regional climate model used in this study are introduced; in Section 3 the results are described and discussed; and finally the summary and conclusions of the work are presented in Section 4.

## Methodology

### 1. Heat stress index

Indices to measure human comfort under given atmospheric conditions have been proposed using a range of variables. Despite the fact that the human body’s response to external conditions depends on individual characteristics (e.g., age, health condition, acclimatization), attempts have been made to quantify the combined effects of different variables on an average person. They are generally based on heat-balance models applied to the human body and range from advanced indices such as the Physiological Equivalent Temperature [27] and the Universal Thermal Comfort Index [28] to more simple metrics based on standard meteorological variables [29]. While more sophisticated indices include wind and radiation factors or physiological variables, in general, they all share the use of temperature and humidity.

To estimate heat stress, we used the simplified Wet-Bulb Globe Temperature (W) developed by the Australian Bureau of Meteorology and its definition can be found in Willett and Sherwood (2010) [22] as:

W=0.567T+0.393e+3.94 (1)

where T and e represent the air temperature (°C) and water vapor pressure (hPa) near the surface. Higher values of W indicate less comfort through increases in either temperature or water vapor. It is based on the Wet-Bulb Globe Temperature index [30], which can only be estimated in very specific locations because it requires non-standard input variables (e.g., Globe thermometer temperature). The simplified version of this metric was chosen because it was designed to use variables that are readily available in most observational datasets such as near surface temperature and vapor pressure. It has also been widely used to measure human comfort [24, 31, 32], which provides a context of heat stress studies in urban and climate change conditions to compare with. Finally, these two variables are well-established risk factors [26] and are known to be affected by the city fabric. The W index is traditionally expressed in °C-equivalent, but in order to avoid any confusion with actual temperature we follow Fischer et al. (2012) [26] and use it as a dimensionless index.

### 2. Model configuration and experimental design

The climate of Greater Sydney Area was simulated at 2-km spatial resolution using the Weather Research and Forecasting [33] modeling system version 3.3.1. The WRF model has been extensively used for both climate studies [34, 35] and urban applications [15, 18, 36]. The performance of the model driven by reanalysis was previously evaluated over the region at temporal scales ranging from daily to inter-annual [37] and configured at 10-km spatial resolution. It has been evaluated at sub-daily timescales [38], and shown to perform adequately. At the resolution chosen in this study, the model was also evaluated extensively [18, 39], and found to perform well overall including the simulation of Sydney’s urban heat island. A more detailed and quantitative description of the model performance through various metrics may be found in these studies.

**Figure 1:**
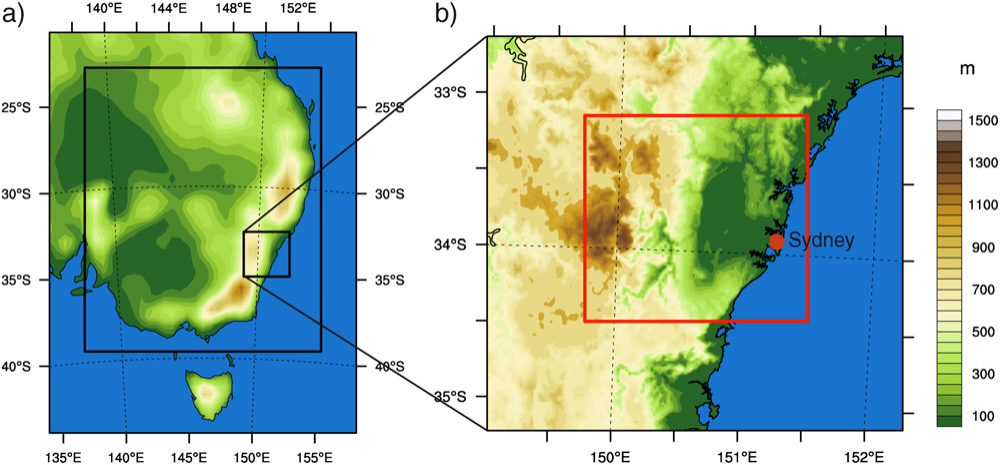
Area of study. a) Topography and location of the coarser 50-km domain. Black lines delimit the extension of the 10- and 2-km domains. b) Topography as described in the 2-km domain over Greater Sydney Area.

The CSIRO-MK3.5 GCM [40] was downscaled over southeast Australia at 50- and 10-km spatial resolution (Fig. 1a) in a previous experiment that spanned 1985–2100. Two time slices comprising 20 years each and representing recent past climate (1990–2009) and future climate under a high-emission scenario (A2, 2040–2059) were further downscaled at 2-km spatial resolution. The model outputs at 10-km were then used to generate the boundary conditions for the 2-km domain that covered an area of approximately 300 by 300km (151 by 146 grid points, Fig. 1b).

The regional model configuration for the two outer domains is described in Evans and McCabe (2010) [37]. The physics schemes selected were: the Noah land-surface model, the Monin-Obukhov surface layer similarity, the Yonsei University boundary layer scheme, the Kain-Fristch cumulus physics scheme, the WRF Single Moment 5-class (WSM-5) microphysics scheme, the Rapid Radiative Transfer Model (RRTM) for longwave radiation and the Dudhia shortwave radiation option.

**Figure 2:**
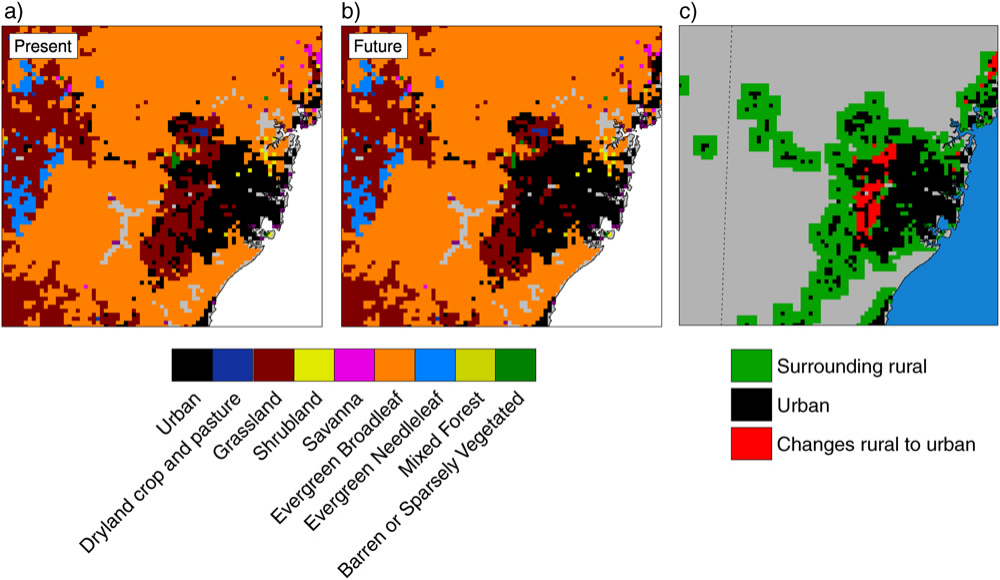
Land use datasets. a) Present dominant land use cover. b) Projected future dominant land use cover. c) Areas of land use change (red)—all changes are from a rural category to urban (new urban). Current extension of the city is represented in black (urban) and a belt of 5 surrounding grid points of rural land cover is represented in green (rural).

A few changes were adopted in the 2-km domain with respect to the coarser domains. The cumulus scheme was switched off assuming that most of the convective processes are explicitly resolved at such resolution. The WSM-5 scheme was replaced with the more sophisticated Thompson microphysics scheme. Finally, sub-grid scale processes in the urban environment were described using the Single-Layer Urban Canopy Model (SLUCM [41]), which unlike more advanced schemes is adequate for spatial resolutions of a few kilometers [42]. The SLUCM uses a tiling approach, where the surface energy budget is calculated separately for both impervious and vegetated cover. These are provided to the atmospheric model according to the percentage of each surface that compose the urban tile, which in this case was set to high-density residential (10% is covered by vegetation). Each grid point is assigned a single land cover category, either urban or one of the natural landscapes (Fig. 2).

The land use of the region is described using a satellite-derived database created by the New South Wales (NSW) Office of Environment and Heritage (OEH) and converted to the 24-category USGS classification to be used in WRF. Two versions of the datasets were created (Fig. 2). A first version representing present land cover in Greater Sydney (OEH-present) and a second one with projected urban expansion (OEH-future). The projected city expansion is based on data from the NSW Department of Planning, which defines areas of future urban development and consolidation for the year 2050. Differences between present and future land uses (Fig. 2c) reflect the urban expansion because all changes are from rural to urban. All urban grid points in the domain were characterized by a high-density residential land-use as defined in the SLUCM default categories.

Three simulations were performed to determine the combined effect of urban expansion and climate change, and compare it with the impact of the climate change signal alone. A first simulation was completed using OEH-present land use and boundary conditions spanning the recent past climate period (1990–2009), and will be referred as control simulation (CTL). A second simulation was run using OEH-present land use and boundary conditions corresponding to the future period (2040–2059), thus incorporating the effect due to climate change alone (CC). Finally, a third experiment was conducted to account for changes due to both climate change and urban development (CC_LU), thus providing future climate (2040–2059) boundary conditions and setting land cover to projected values using OEH-future land use.

The contribution of urban expansion to projected changes in the local climate was quantified by comparison of the two future climate simulations. The changes due to climate change alone described in CC are compared to projections from CC_LU, which included both climate change and urban expansion. Both phenomena—climate change and urban expansion—interactions are non-linear and their effects cannot be regarded as independent and additive [11, 12]. Therefore the differences between CC and CC_LU reflect the sum of effects coming from urbanization itself and the coupling of climate change and urban development.

Changes are calculated using the present climate control run (CTL) as reference. Since the response of the local climate to urban influence has a marked diurnal cycle, being generally stronger at night [4, 18, 43], changes of maximum and minimum W are calculated separately to study responses at day and night. An accurate estimation of W daily extremes requires high-frequency outputs and W must be computed using simultaneous relative humidity and temperature [26]. Model outputs were saved at hourly frequency for all near-surface standard variables to ensure a good approximation of the actual maximum and minimum W in the model. For brevity, the times when W reaches its daily maximum (minimum) will be referred as day (night) values.

Temperature and humidity changes are discussed first and their aggregated contribution to heat stress is then examined. Temperature and vapor pressure were also extracted at the time of maximum and minimum W as representative of day and night values.

## Results

### 1. Temperature and humidity

**Figure 3:**
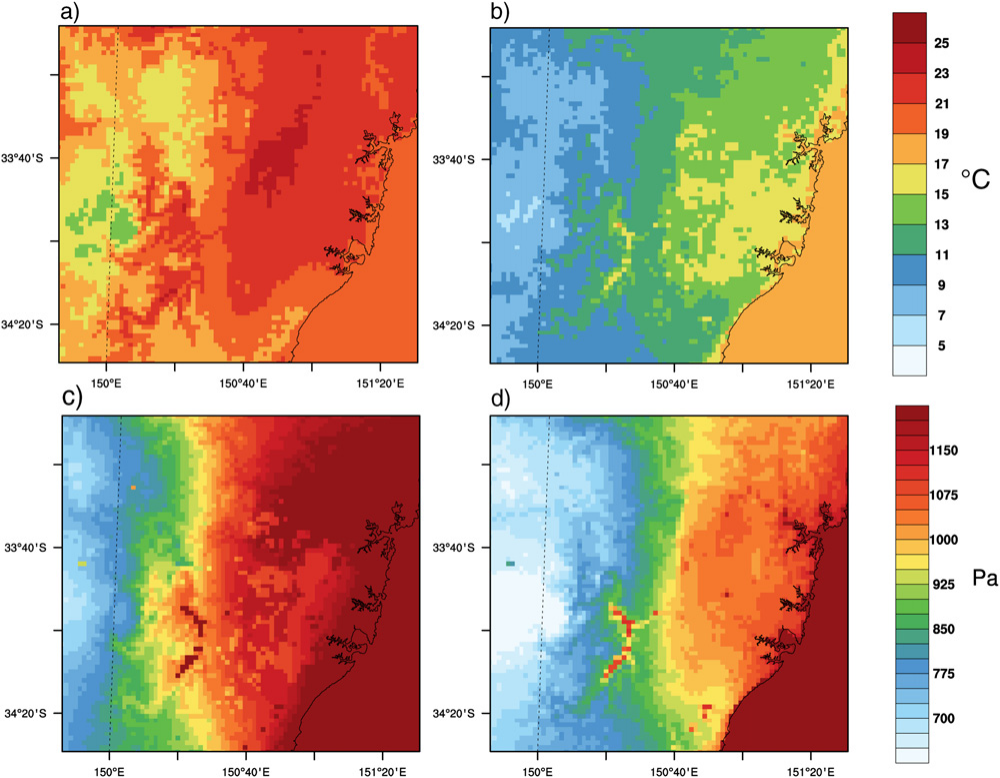
Temperature and vapor pressure climatology. Annual mean temperature at time of daily maximum (a) and minimum (b) W. Annual mean vapor pressure at the same times (c, d). Both from present climate (1990–2009) run.

Annual mean values of temperature (*T*) and vapor pressure (*e*) at day and night are shown in Fig. 3 to provide a context for the changes analyzed later. Temperature at the time of maximum W (Fig. 3a) is strongly driven by topographical features, and the presence of the city does not have a clear influence on its annual mean spatial pattern. However, at the time of minimum W (Figs. 3b) temperature presents a weaker dependency on elevation and a marked response to the presence of urban areas. Indeed, the boundaries of the city are identifiable in the spatial pattern of annual mean nighttime temperature, with urban areas showing higher temperatures (yellow) than the surroundings.

Unlike temperature, vapor pressure shows a stronger response to the presence of the city during the day, but not during the night (Figs. 3c–d). There is also a dependency on the distance to the ocean observed both at day and night. The extension of the city is clearly identifiable in the spatial patterns of vapor pressure at the time of maximum W, because *e* is generally smaller in urban areas. This is consistent with our current knowledge of the energy processes and the surface flux partitioning in cities [2]. The combined contrasts of temperature and vapor pressure between urban and rural areas shown in Fig. 3 lead to relative humidity values at night 10–15% drier (not shown) in the urban environment than in the rural one, which is also consistent with previous finding using GCMs [26].

**Figure 4:**
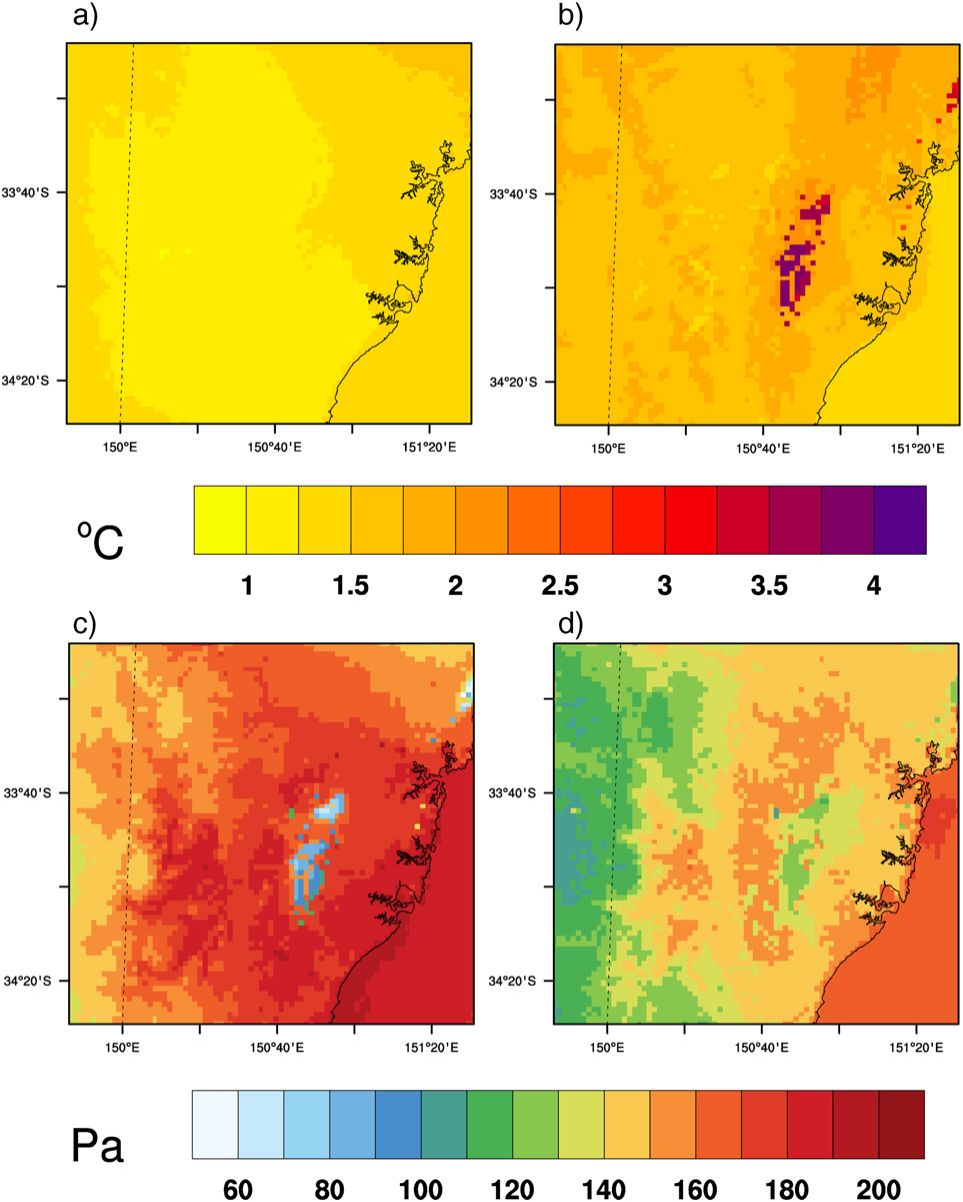
Temperature and vapor pressure changes. Annual changes in 2-m temperature at the time of maximum (a) and minimum (b) W. Annual changes in 2-m vapor pressure at the time of maximum (c) and minimum (d) W. Results from the combined effects of climate change and urban expansion (CC_LU-CTL).

Fig. 4 illustrates the annual mean changes of temperature and vapor pressure between CTL and CC_LU simulations at the time of maximum and minimum W. Changes in temperature during the middle hours of the day, when W usually reaches its maximum are mostly affected by the large-scale climate change signal and nearly no effect of urban expansion is detected in maximum temperature. Indeed changes from CC_LU (Fig. 4a) and CC (S1 Fig.) simulations are almost identical and the magnitude of the changes under the selected future climate conditions range between 1.0 and 2.0°C across the domain in both runs.

On the other hand, urbanization has a noticeable effect on changes in temperature at the time of daily minimum, which generally occurs at night or near sunrise. In this case, the new urban areas leave a clear footprint in the annual mean changes according to CC_LU run (Fig. 4b), for which both climate change and urban development were considered. Such a footprint is not obtained in the CC run (S1 Fig.). While most of the domain will be subjected to changes in nighttime temperatures within the range 1.5 to 2.5°C according to both CC and CC_LU, the latter projects increases up to 3.5 to 4.0°C in new urban areas. Changes in new urban areas within the Sydney metropolitan region are twice the changes when no urban expansion occurs.

In a warmer climate, a moister atmosphere is expected [22]. However, while the city tends to amplify future changes in temperature at local scales, at least during the coolest hours of the day, the effect of urban surfaces is that of partly counterbalancing the projected increase in humidity, particularly during the day (Figs. 4c–d). Both future simulations (CC and CC_LU) produce higher values of vapor pressure over the entire domain, but such increases are substantially smaller over areas of urban expansion in the CC_LU run (Figs. 4c–d). During the day, increases in the range of 150–190 Pa are projected for vapor pressure in the Sydney area according to both CC (S1 Fig.) and CC_LU (Fig. 4c), but CC_LU projects increases in vapor pressure between 60 and 100 Pa over new urban areas. The combined effect of climate change and urbanization also leads to more moderate increases in vapor pressure at night (120–130 Pa) compared to changes in the range 140–160 Pa obtained from the CC run, although the contrast is not as marked as during the day.

Overall, climate change and urban expansion both act on temperature and humidity, but the outcome of their effects on each variable contribute to heat stress changes in opposite directions and at different times of the day. When W reaches its highest daily peak, urbanization primarily reduces vapor pressure increases, while it enhances warming when W drops to its daily minimum.

### 2. Simplified wet-bulb globe temperature

Temperature and humidity are combined in W (Equation 1) to measure human comfort and to determine the impact of urban expansion and climate change on local heat stress. We also considered the use of the Apparent Temperature [29] to incorporate possible urban-induced changes of wind speed but it was found that urban expansion in this region has a minor impact on light wind regimes and no discernible effect on stronger winds [18]. Indeed, results using Apparent Temperature give higher relative importance to temperature but lead to similar conclusions and thus W was selected for its simplicity.

In addition to wind, radiation changes could also affect daily maximum heat-stress changes. Only minor increases in downward radiation during central hours of the days are projected due to the greenhouse gas effect, and no impact from urban areas is detected (S2 Fig.). Therefore, it is reasonable to assume that heat-stress changes are mainly explained by temperature and humidity changes, with radiation playing only an indirect role through temperature increases.

In the previous section, it was found that changes in temperature and humidity may counterbalance and analyzing temperature changes alone could lead to inaccurate conclusions in terms of impact on population. In this section the relative importance of the urban-induced changes in these variables is quantified. Unlike temperature and humidity, W is examined at seasonal timescales to better investigate the responses throughout the year.

Mean seasonal values of daily maximum W range between 10 in the high-elevation locations of the domain during winter (JJA) and 32 over lowlands in summer (DJF). As for the daily minimum seasonal means, W over land ranges between 6 and 26 (S3 Fig.) with the lowest values attained during winter in the interior and the highest values along the coast in summer. Heat-stress contrast between urban and surrounding rural areas in CTL simulation indicate that maximum W is on average approximately 0.7 larger in the city than in the surrounding areas (Table 1). Urban land effect on heat stress is more prominent at the time of minimum W, when differences between urban and neighboring rural areas are on average 2.1. Autumn (MAM) and spring (SON) show the largest differences for minimum W, whereas maximum W contrasts are more pronounced in autumn (MAM) and winter (JJA).

**Table 1:** Present-climate heat stress values over rural and urban areas.

		**Summer (DJF)**	**Autumn (MAM)**	**Winter (JJA)**	**Spring (SON)**	**Annual**
Minimum W	Rural	21.0	16.8	10.4	14.4	15.6
	Urban	22.9	18.8	12.3	16.7	17.7
Maximum W	Rural	26.1	21.5	15.6	20.6	20.9
	Urban	26.6	22.3	16.5	21.2	21.6

Present-climate seasonal and annual means of minimum and maximum W averaged over all urban grid points in the domain (urban) and over a belt of 4 adjacent land points (rural) to any urban tile (Fig. 2c), from CTL simulation.

Urban and rural contrasts under present climate conditions suggest that temperature and humidity changes induced by the presence of the city lead to more intense heat stress conditions both at day and night, although the effect is considerably larger on minimum W. The question that arises is whether urban expansion will play a similar role in shaping heat stress in a changing climate.

**Figure 5:**
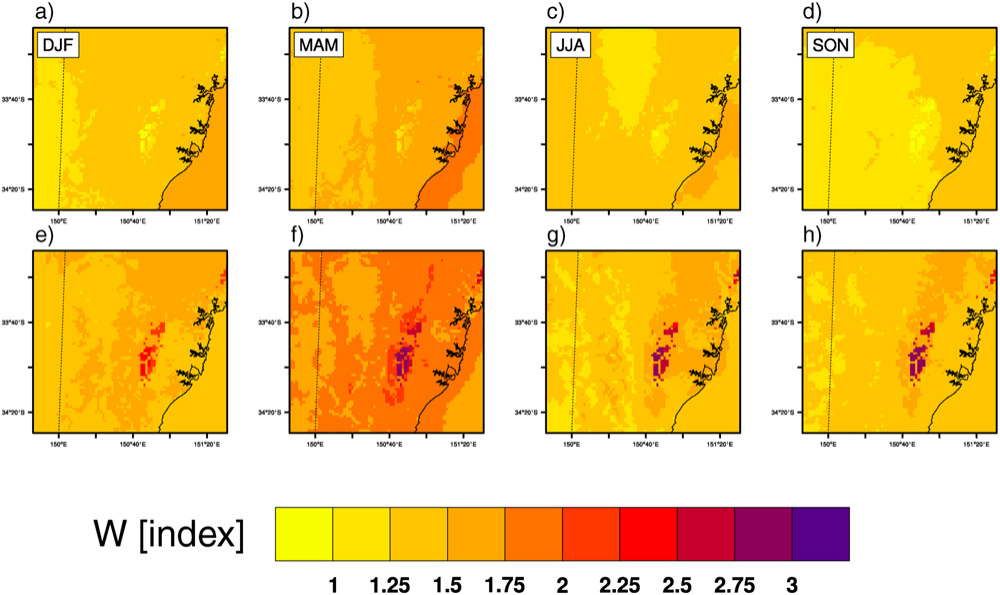
Daily maximum and minimum heat stress seasonal changes. Seasonal changes of daily maximum (a–d) and minimum (e–h) W due to both climate change and urban expansion (CC_LU-CTL).

Fig. 5 illustrates spatial patterns of W seasonal changes from CC_LU simulation. Changes due to climate change alone (CC run) are shown in S4 Fig. Changes in maximum W (Figs. 5a–d) are generally smaller than changes in minimum W (Figs. 5e–h) across the domain. In general, the combined increases in *T* and *e* projected both during day and night (Fig. 4) result in larger W changes at night, suggesting that temperature changes dominate over humidity changes.

In addition to projections over the entire domain where climate change is acting alone, the clearest feature of W seasonal changes is that new urban areas will significantly contribute to daily minimum heat stress values. This again emphasizes that vapor pressure contrasts between urban and rural areas are not enough to offset the warmth induced by urban areas.

**Figure 6:**
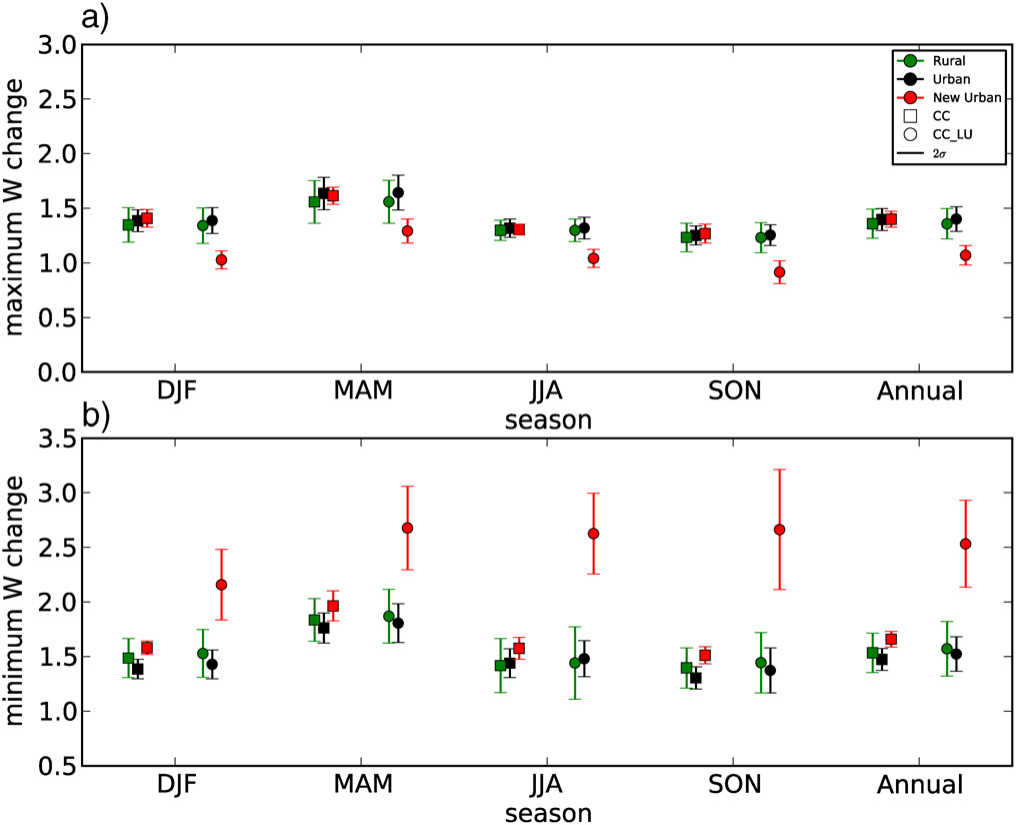
Seasonal and annual W changes in rural, urban and new urban areas. Area-averaged seasonal and annual changes of mean daily maximum (a) and minimum (b) W over present urban areas (black), future urban expansion areas (red) and city surrounding belt of rural areas (green). CC (CC-CTL) simulation is represented with squares and CC_LU (CC_LU-CTL) with circles. The error bars represent 2 standard deviations of the changes over all grid points within each area as a measure of the spread.

Three different regions were identified in the domain to represent existing rural and urban areas that will remain in the future, and rural areas that will change to urban. They are labeled urban, rural and new urban respectively (Fig. 2c). Seasonal changes over the three different areas were calculated to summarize the impact of urbanization on mean daily maximum and minimum W (Fig. 6). Differences between future runs over areas of potential land use change show that urbanization contribution at night is particularly marked during winter and spring, when new urban areas will be exposed to W increases of more than 2.5, whereas changes over the same areas but without urban expansion (CC run) are projected to be just below 1.5. A similar pattern is observed for summer and autumn, although the contrast between CC and CC_LU is not as pronounced as for the other two seasons. Interestingly, urbanization and climate change acting together results in more moderate increases in daily maximum W than if only climate change is considered (Fig. 6a). This indicates that although W changes are generally dominated by temperature changes, urban-induced changes in vapor pressure are important during the day and partially compensate for W increases due to future warming.

From the human comfort perspective, changes in extremes may be more important than changes in the mean. Seasonal changes for both the 95^th^ and the 99^th^ percentile of daily maximum and minimum W were also examined (not shown) and the spatial patterns obtained were similar to their respective seasonal means. Similarly to changes in the means, area-averaged changes over representative regions shown in Fig. 2c were calculated for the seasonal 99^th^ percentile of maximum and minimum daily W (S5 Fig.). Changes in extreme maximum W are smaller in areas of expected city sprawl when incorporating both land use changes and climate change effects (CC_LU) than in the simulation considering only the latter (CC). Regarding changes in nights of particular discomfort, minimum W will increase more when urban development and climate change are considered together, but differences between CC and CC_LU are not as marked as for the means. Taking 2σ (twice the standard deviation) over all grid points within each of the three areas as a measure of the spread in the changes, it is worth noting that only the range of annual maximum W change in new urban areas is outside the range of spread of the other two regions (rural and urban).

**Figure 7:**
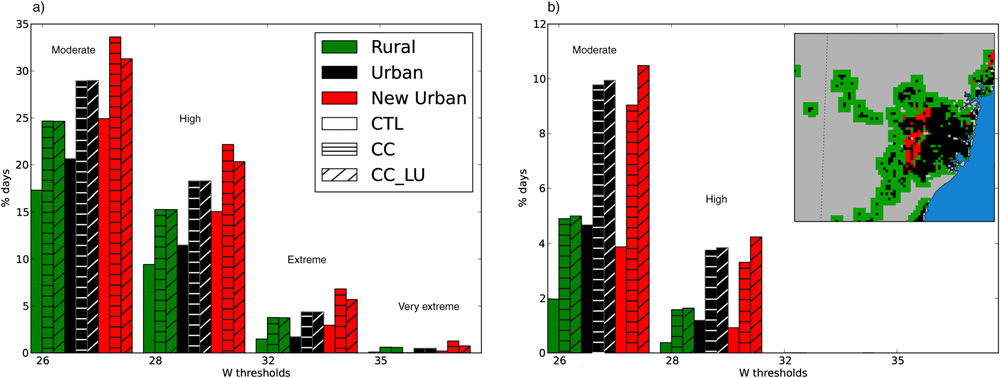
Present and future frequency of adverse conditions caused by heat stress. Percentage of days when daily maximum (a) and minimum (b) W exceed different thresholds in the surrounding rural (green), urban (black) and new urban (red) areas as defined in the top right map. Probability for each of the areas is calculated according to CTL (plain), CC (horizontal striped) and CC_LU (diagonal striped) runs.

Fig. 7 shows the percentage of days/nights when W exceeds various thresholds that were proposed to assess different levels of human vulnerability to heat stress [22, 26], similarly to Oleson et al. (2013) [44]. They provide an estimation of population exposure to heat stress moderate (26), high (28) and extreme (32) risk, and are most relevant for healthy people doing physical activity outdoors. A fourth threshold (35) was also added to quantify the occurrence of very extreme conditions, which corresponds to the human physiological limit referred to in Sherwood and Huber (2010) [32]. The frequency of thresholds exceedance was calculated for each of the identified areas within the domain separately. The percentage of days above each of the thresholds in the CTL run is quite different in the three areas, with areas of potential urban expansion showing a particularly high number of days/nights above the moderate and high thresholds (also above the extreme threshold for daytime W). This shows that there are differences among these areas during present climate conditions that cannot be attributed to land use only, and other factors such topography are also in play.

Changes in maximum W thresholds exceedance are similar in all three regions when climate change is acting alone (CC) compared to their respective present climate values. However, our results also suggest a larger number of days above the moderate- and high-risk thresholds in urban areas (black) than in the rural surroundings (green) in all runs. Therefore, W will be more likely to exceed these thresholds in the city both in the present and in the future. The contribution of land use change in new urban areas (red) reduces the impact due to climate change alone and the percentage of days above each of the thresholds is smaller in CC_LU than in CC. No effect is detected beyond areas of city sprawl since CC and CC_LU project very similar changes in the other two areas. In relative terms, GHG-induced changes tend to be more pronounced as we move to higher-risk W values. For instance, the number of days with maximum W within the extreme zone could double according to both future projections and over all areas, although urban expansion contribution partly offsets the climate change signal during daytime.

Nighttime W shows a stronger response to future changes (Fig. 7b). Future projections suggest more than double the number of nights when W will exceed both moderate- and high-risk thresholds compared to present values. The increase is particularly prominent for areas of city growth (red) since climate change and urban expansion will lead to changes from below 4% to over 10% of the nights above 26, and from less than 1% to more than 4% of the nights exceeding the high-risk threshold. Despite the fact that urban climate change remains the main contributor to changes in particularly adverse nighttime conditions, urban expansion further enhances the probability for those conditions to occur.

**Figure 8:**
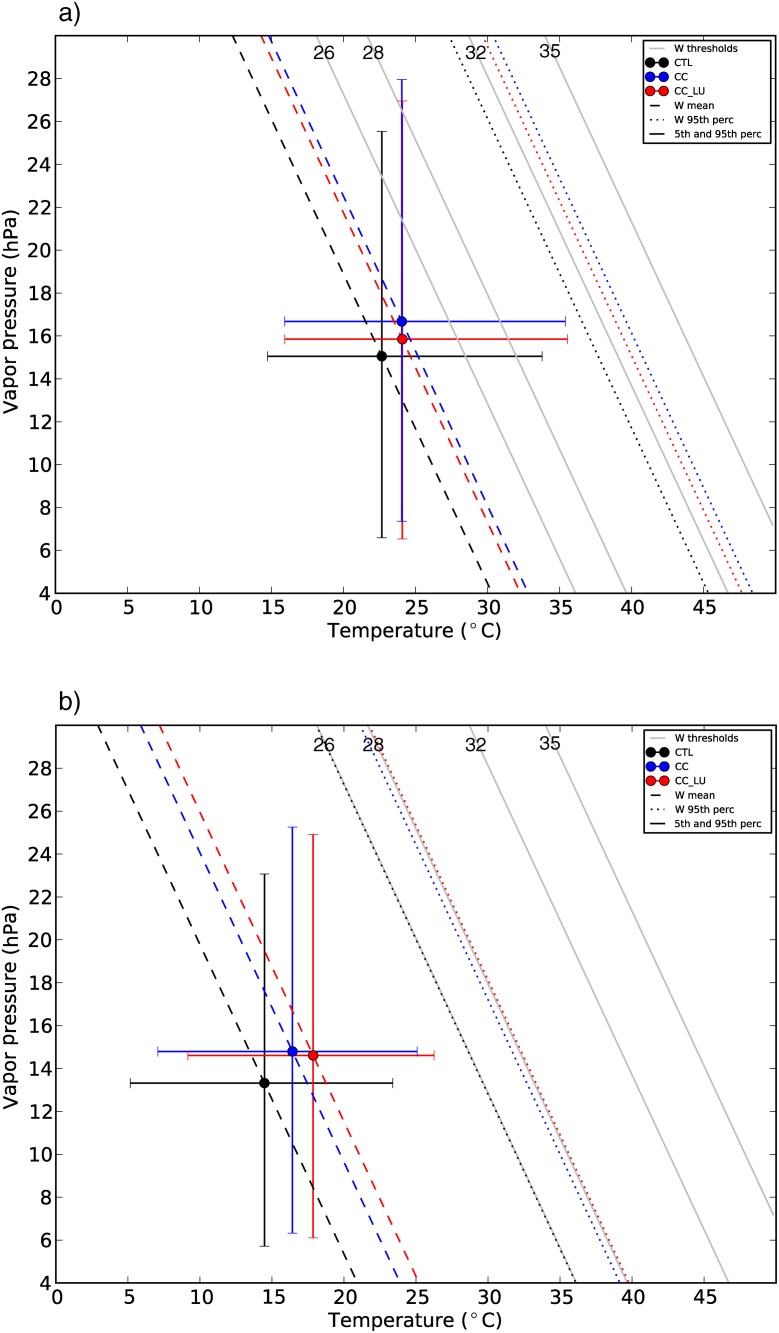
Relationship between vapor pressure, temperature and W. Vapor pressure versus temperature plots at the time of daily maximum (a) and minimum (b) W. Mean values of vapor pressure and temperature are represented with circles for the three simulations (CTL, black; CC, blue; CC_LU, red). The bars represent the 5^th^ and 95^th^ percentiles of vapor pressure and temperature. The gray lines represent the W thresholds 26, 28, 32 and 35 for any combination of vapor pressure and temperature within the range. Mean values (dashed lines) and 95^th^ percentile (dotted lines) of W are also shown for all three simulations.

The way changes in vapor pressure and temperature impact future W is shown in Fig. 8, which is divided into values at the time of daily maximum (a) and minimum (b) W. All values of vapor pressure and temperature are represented for each of the simulations over the areas of expected city growth. For clarity, all possible values are summarized in these plots showing some statistical properties of the group of data points. Circles represent mean vapor pressure versus mean temperature and the bars represent the 5^th^ and the 95^th^ percentile for each of the variables so that the spread is visualized. Dashed and dotted lines represent the W mean and the 95^th^ percentile respectively calculated over all grid points within the new urban areas and all days.

Fig. 8 illustrates the idea that during daytime, urban development induces lower humidity compared to the run without land use changes (blue). Temperature is very similar in both CC and CC_LU simulations and its projected changes in both the mean and tails of the distribution (5^th^ and 95^th^ percentiles for each grid point within the new urban areas) are almost indistinguishable. These changes lead to an environment with overall lower W values, as represented by the dashed and dotted lines in Fig. 8a. Humidity deficit produced by new urban areas compared to climate change effects is thus responsible for differences in W between both future runs. Nevertheless, differences between CC and CC_LU remain small compared to the magnitude of the climate change signal effects in terms of both mean and extreme (95^th^ percentile) W.

At night, temperature substantially differs between CC and CC_LU over new urban areas (Fig. 8b), whereas increases in humidity are very similar in both simulations. Despite humidity changes weakly compensating temperature increases, the nighttime change in temperature in new urban areas is large enough to override any vapor pressure difference with CC and enhance the projected increases in minimum W due to climate change alone.

In terms of mean values of maximum and daily minimum W, urban expansion increases the effects of the chosen climate change on minimum W by approximately 50% at night, whereas during the day, urban impact is smaller compared to the climate change effect. It is interesting to note that the gap between CC and CC_LU mean minimum W values is larger than for the extremes (95^th^ percentile), but the contrary occurs for maximum W.

**Figure 9:**
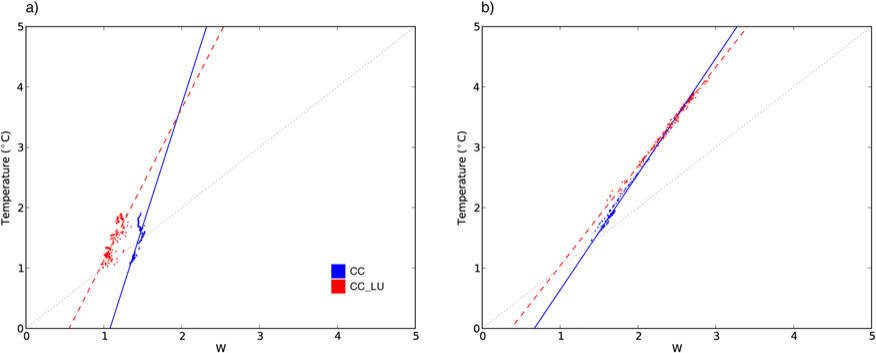
Relationship between temperature and W changes. Annual changes of temperature versus annual changes of W at the time of maximum (a) and minimum (b) W. Each dot represents annual mean changes for each grid point within the area of potential urban expansion according to simulations including only climate change (CC-CTL, blue) and both climate change and urban expansion (CC_LU-CTL, red). Lines represent linear fits of the points using least squares.

Fig. 9 illustrates the relationship between projected annual changes in temperature and W in areas subjected to urban expansion for both CC and CC_LU simulations. It is noteworthy how linear the correspondence between temperature and W annual changes at night is, and indeed correlations with the linear fitting are above 0.98 for both runs. During daytime, such a relationship is not as clear and correlation actually drops, although it is still above 0.80. Linear correlations were also calculated at seasonal scales obtaining values above 0.97 for all seasons and runs at the time of minimum W, and lower values at the time of maximum W, especially in autumn when correlations are as low as 0.59 for CC and 0.57 for CC. This further emphasizes which of the two variables defining W plays a dominant role in each of the W extremes. Another interesting feature is that at night, the relationship between temperature and W changes is very similar in both future simulations (Fig. 9b), whereas during the day, the effect of temperature changes are not reflected in W changes equally for CC and CC_LU. Nonetheless, for both extremes, the slope that relates changes in both variables tends to be higher for CC, especially during the day. As such, red and blue points are closer together at the top end and despite a weaker response of W to higher temperatures in both cases, it suggests that urban expansion could contribute to enhance maximum W changes under a stronger climate change signal. However, the highly non-linear nature of the interactions in the climate system precludes any simple extrapolation of the results. Instead, these results highlight the importance of quantifying the effect of urbanization under stronger warming by either choosing a different emission scenario or examining a period when the climate change signal is larger.

## Summary and Conclusions

An analysis of the combined effect of urban expansion and climate change on heat stress over the Sydney Area was conducted using a regional climate model with explicit representation of the urban environment. Two simulations of a plausible future climate were performed, with the only difference being the urban land expansion. These were compared with a present climate run to quantify the effect of city sprawl under climate change conditions. Following previous studies [22, 24] the combined changes in temperature and humidity were examined using the simplified wet-bulb globe temperature to quantify human heat-stress and determine the contribution of urban development to human discomfort under a future climate scenario.

The effects of urban areas on local temperature and vapor pressure from the simulations are in agreement with previous results [4, 23] and with the theoretical basis of energy partitioning in the urban environment [21]. Replacing rural land surfaces by urban ones in the future contributes to nighttime temperature changes and leads to further warming when compared to the effect of increased greenhouse gas concentrations alone. The impact of the city sprawl on daytime temperature is negligible compared to the large-scale increases induced by greenhouse gases. New urban areas affect humidity during both day and night. During the central hours of the day, the effect of urbanization compensates to some extent the overall increase of humidity produced by global warming. As a result, in areas of expected city growth, climate change and urbanization together produce changes in vapor pressure substantially smaller than climate change alone. Therefore, changes in daily maximum W are projected to be slightly smaller when urban development is considered. In any case, the occurrence of risk conditions will substantially increase in the entire region and, indeed, days with heat stress exceeding the top-risk thresholds (32 and 35) could double present climate values. Both existing and future urban areas will be more likely exposed to moderate- (26) and high-risk (28) heat-stress conditions. Only new urban areas are projected to experience a larger number of days with particularly adverse conditions (32 and 35) compared to the rural areas. At night, increased heat storage of city structures and the following release after sunset produces much higher temperature changes than in the equivalent rural areas. Such an increase is comparable to the magnitude of the climate change signal at local scales and is not counterbalanced by decreases in humidity projected for new urban areas. Hence, temperature changes at night dominate changes in heat stress, which will markedly increase in areas where the city is expected to grow. Nights of moderate heat stress (26) will double in the future and those with high heat-stress (28) will triple. Although changes in threshold exceedance are similar in rural and urban areas, the latter will experience a higher probability of encountering nighttime heat-stress conditions. Here we focused on the occurrence of particularly adverse conditions, although the prolonged exposure to such conditions might also be relevant from a human perspective. An interesting continuation of this study is to determine the persistence of heat stress conditions to further assess impacts on population.

In contrast to previous studies [26], the upper tail of minimum W distributions are not as influenced by urbanization as the mean. On the other hand, extremes of maximum W are more affected by urban expansion than the mean. Differences in the high percentiles of temperature and humidity are responsible for this contrasting response.

In this study, we have shown the inadequacy of estimating seasonal heat stress changes using temperature alone and the need to incorporate the effect of humidity, particularly in the assessment of daytime values. The relationship between changes in temperature and W also suggests that the effect of urban expansion under stronger climate change signal should be examined because the impact of city sprawl could be different under such scenarios.

This work has not included the effect of sources of heat and water vapor coming from human activity, which could worsen heat stress conditions in the city by increasing both temperature and humidity. The effect of progressive city densification was not considered either and only a single high-density residential category was assigned to every urban grid cell. Future work will sample the range of possible future scenarios in terms of both climate and urban development.

## Supporting Information

S1 Fig Temperature and vapor pressure changes due to climate change. Annual changes in 2-m temperature at the time of maximum (a) and minimum (b) W. Annual changes in 2-m vapor pressure at the time of maximum (c) and minimum (d) W. Results from the effects of climate change alone (CC run).
(EPS)

S2 Fig Daytime downward radiation changes. Changes in downward components of radiation (longwave plus shortwave) at 16H local time projected by CC run (a) and CC_LU (b) compared to the present climate run (CTL). Changes are expressed in percentage of the CTL run estimates.
(EPS)

S3 Fig Seasonal climatology of daily maximum and minimum W. Seasonal mean values of daily maximum (a–d) and minimum (e–h) W from present climate simulation (CTL) over the period 1990–2009.
(EPS)

S4 Fig Seasonal changes of W due to climate change. Seasonal changes of daily maximum (a–d) and minimum (e–h) W due to climate change alone (CC run).
(EPS)

S5 Fig Changes in the W 99^th^ percentile over rural, urban and new urban areas. Area-averaged seasonal and annual changes of the 99^th^ percentile of daily maximum (a) and minimum (b) W over present urban areas (black), future urban expansion areas (red) and city surrounding belt of rural areas (green). CC (CC-CTL) simulation is represented with squares and CC_LU (CC_LU-CTL) with circles. The error bars represent 2 standard deviations of the changes over all grid points within each area as a measure of the spread.
(EPS)
